# Short-term perinatal oxygen exposure may impair lung development in adult mice

**DOI:** 10.1186/s40659-020-00318-y

**Published:** 2020-11-10

**Authors:** Vasantha H. S. Kumar, Huamei Wang, Lori Nielsen

**Affiliations:** Division of Neonatology, Department of Pediatrics, University At Buffalo, 1001 fifth Floor Main Street Buffalo, Buffalo, NY 14203 USA

**Keywords:** Oxygen, Gene expression, Cell cycle, Lung, Resuscitation

## Abstract

**Background:**

Hyperoxia at resuscitation increases oxidative stress, and even brief exposure to high oxygen concentrations during stabilization may trigger organ injury with adverse long-term outcomes in premature infants. We studied the long-term effects of short-term perinatal oxygen exposure on cell cycle gene expression and lung growth in adult mice.

**Methods:**

We randomized mice litters at birth to 21, 40, or 100%O_2_ for 30 min and recovered in room air for 4 or 12 weeks. Cell cycle gene expression, protein analysis, and lung morphometry were assessed at 4 and 12 weeks.

**Results:**

The principal component analysis demonstrated a high degree of correlation for cell cycle gene expression among the three oxygen groups. Lung elastin was significantly lower in the 100%O_2_ groups at 4 weeks. On lung morphometry, radial alveolar count, alveolar number, and septal count were similar. However, the mean linear intercept (MLI) and septal length significantly correlated among the oxygen groups. The MLI was markedly higher in the 100%O_2_ groups at 4 and 12 weeks of age, and the septal length was significantly lower in the 100%O_2_ groups at 12 weeks.

**Conclusion:**

Short-term exposure to high oxygen concentrations lead to subtle changes in lung development that may affect alveolarization. The changes are related explicitly to secondary crest formation that may result in alteration in lung elastin. Resuscitation with high oxygen concentrations may have a significant impact on lung development and long-term outcomes such as BPD in premature infants.

## Background

An abrupt transition of the fetus from a relatively hypoxic to a relatively hyperoxic environment results in physiologic oxidative stress in infants soon after birth [1]. Due to the lack of induction of antioxidant enzyme (AOE) systems [2], premature infants are particularly sensitive to the toxic effects of oxygen. Resuscitation with 100%O_2_ generates oxygen radicals [3], and subsequent reperfusion injury may further exacerbate free radical production and oxygen toxicity [4]. Furthermore, supplemental oxygen contributes to the development of bronchopulmonary dysplasia (BPD) [5], retinopathy of prematurity (ROP) [6], and brain injury [7] in premature infants. However, there is uncertainty as to whether initiating resuscitation post-birth with lower (FiO_2_ < 0.40) or higher (FiO_2_ ≥ 0.40) in the first ten minutes of birth has on mortality, morbidity, and other long-term outcomes in premature infants [8, 9]. Current neonatal resuscitation guidelines recommend initiation of resuscitation with low oxygen (FiO_2_: 0.21–0.30) and to titrate the oxygen concentration to achieve preductal oxygen saturation approximating the interquartile range measured in healthy term infants [10]. Due to concerns about oxidative injury, the guidelines do not recommend initiation of resuscitation with ≥ 65%O_2_ [10]. The optimal oxygen concentration required at resuscitation of premature infants is one of the contentious issues in newborn care.

We have demonstrated that ventilated premature lambs cannot appropriately increase antioxidant activity in response to hyperoxia, and that increased exposure to O_2_ aggravates oxidant lung injury in these lambs [11]. A brief period of high oxygen concentrations led to increased pulmonary arterial contractility at 24 h of newborn lambs [12]. Reactive oxygen species (ROS) from excess oxygen exposure influence the molecular and biological processes observed in cells during differentiation and aging [13]. Furthermore, these processes are affected by changes in gene expression and signal transduction that act as messengers for growth factors [14]. Because oxidative stress influence apoptosis and cell growth [15], hyperoxia may have long-term consequences on growth & development. Although the relationships among the stage of the cell cycle, redox state, and oxidant production are poorly understood, mitotic progression influences the redox state and vice versa [16, 17]. The mitotic index of the postnatal lung is significantly higher compared to the adult lung, and perinatal oxygen exposure may alter cell cycle gene expression, mitotic progression, and hence cell proliferation [18, 19].

Hyperoxia at resuscitation also increases systemic oxidative stress and oxidative lung injury [20]. Brief exposure to high oxygen concentrations during stabilization may trigger organ injury with adverse long-term outcomes in premature infants [21]. We studied the effects of oxygen exposure soon after birth on cell cycle gene expression and lung alveolarization in adult mice. We studied three different concentrations of oxygen (21%O_2_, 40%O_2,_ and 100%O_2_) in an experimental model of resuscitation in newborn mice to assess long-term effects on the lung at four weeks and 12 weeks in adult mice. We hypothesize that a brief period of exposure to 100%O_2_ in the perinatal period may alter cell cycle gene expression with implications on lung alveolarization in adult mice.

## Methods

### Oxygen exposure

We performed an in vivo study of short-term exposure to O_2_ in newborn mice to mimic the exposure to hyperoxia during the resuscitation of premature infants after IACUC approval of the University at Buffalo (Project # PED24116N). Time**-**dated pregnant C57Bl/6 mice were acclimatized in the lab animal facility after purchasing from the vendor (Envigo RMS Inc, Indianapolis, IN) before delivery. Dams were observed on the day of expected delivery (q3h) with minimal disturbance. We had 16 dams delivering 80 pups (average of six pups/dam) with a mortality of 10% (8 pups) during the study. Oxygen exposures were performed as close to birth as possible and no later than 6 h of age. Litters were allocated by simple randomization using cards to one of the three groups (21%O_2_, 40%O_2,_ or 100%O_2_). Four-week experiments were performed initially, followed by twelve-week mice experiments. Animal cages were placed in a Plexiglas chamber pre-filled with 100%O_2_ or 40%O_2_ and covered with a plastic wrap. Oxygen concentration was maintained in the chamber both before and during the experiment with ProOx 110 oxygen controller (BioSpherix, NY). ProOx-110 senses oxygen concentration inside the chamber and infuses either oxygen or nitrogen, respectively, to increase or decrease the oxygen concentration in the chamber. The chamber was monitored continuously during the 30 min of oxygen exposure to confirm the precise administration of oxygen concentrations into the cages. Humidity (50–60%) and the temperature was identical to all exposures. Mice in 21%O_2_ were subjected to an identical environment as hyperoxia-exposed mice.

All mice litters were recovered in room air (RA) after 30 min of oxygen exposure. Mice were sacrificed at 4 or 12 weeks of age by intraperitoneal injection of sodium pentobarbital. Gene expression and protein analysis were performed on frozen lung tissue in all the three O_2_ groups at 4 and 12 weeks of age (N = six in each group, each time point). Formalin studies were performed in a separate set of mice at both time points (N = six in each group, each time point).

### RNA isolation

RNA was isolated from flash-frozen mouse lung using the RNeasy Mini kit (Qiagen, Valencia, CA) with on-column DNase digestion per manufacturer's protocol. RNA integrity was assessed using the Experion Automated Electrophoresis System (BioRad, Hercules, CA).

### Whole lung gene expression profiling by RT^2^-qPCR

The cell cycle PCR array (Mouse Cell Cycle RT^2^ Profiler PCR Array; SA Biosciences, MD) profiles the expression of 84 specific genes that regulate the cell cycle. The PCR array performs gene expression analysis with real-time PCR sensitivity and the multi-gene profiling capability of a microarray. Using the RT^2^ first strand kit (SA Biosciences, MD), 300 ng of total RNA was reverse transcribed to cDNA, mixed with RT^2^-SYBR Green qPCR master mix. Aliquots of this mix were placed into each of the PCR array plates containing the predispensed gene-specific primer sets. PCR performed on a 96 well MyiQ thermocycler (BioRad, Hercules, CA) according to the manufacturer's protocol. The threshold cycle (C_t_) values and the fold change in gene expression for pair-wise comparison were processed using the excel-based PCR Array Data Analysis software (SA Biosciences) applying the equation 2-∆∆C(t) by comparing to the corresponding RA group (21%O_2_-4 weeks; 21%O_2_-12 weeks). We used three housekeeping genes (Glyceraldehyde-3-phosphate dehydrogenase (*Gapdh*), Hypoxanthine guanine phosphoribosyl transferase (*Hprt*) & Actin, beta (*Actb*) based on stability between experimental conditions.

### Cell cycle protein analysis

Cdkn1a (p21), a cell cycle inhibitor; Ki67, a marker of cell proliferation; tumor protein 63 (Trp63), a protein that governs tissue morphogenesis; cyclin B1 (CCNB1), a protein that is essential for the control of cell cycle at the G2/mitosis transition and elastin (ELN), a necessary protein for alveolar development were analyzed in frozen lung tissue. Tissues were pulverized on dry ice, suspended in PBS with protease inhibitors, spun at 16,000 *g* for 3 min, and the supernatant used for cytoplasmic protein ELISAs (p21, Elastin, Cyclin B1). The pellet was suspended in PBS and centrifuged × 2 (500 *g* for 15 min; 1000 *g* for 15 min), discarding the supernatant each time. The pellet was resuspended in buffer (20 mM HEPES;1%Triton-x-100), and nuclei lysed by passing the suspension through an 18 g needle 20 times; spun at 9000 g for 30 min. The resulting supernatant (nuclear protein) was used for the determination of Ki67 and Trp63 proteins by ELISA. Protein concentration for both fractions was determined by the BioRad DC Protein assay (Hercules, CA). ELISAs were performed according to the manufacturer's protocol. Mouse Trp63, MKi-67, Cdkn1A, and Cyclin B1 ELISAs obtained from MyBioSource (San Diego, CA) and Elastin ELISA from Elabscience (Wuhan, China).

### Histopathology

The trachea was cannulated, and the lungs were instilled with 10% buffered formalin at 25 cm of H_2_O pressure. The lungs were embedded in paraffin, cut into 5 µm thick sections, and stained with hematoxylin–eosin and elastin (Verhoeff's elastin stain). Lung morphometry was assessed in twenty representative images per lung section and five sections per mouse from different regions of the lung (six animals per group; three groups each at 4 and 12 weeks). Lung morphometry measurements were performed at 200 × resolution in all groups (850 × 450 µm; 382,000 µm^2^). Alveolization was estimated by the radial alveolar count (RAC) method of Emery & Mithal [22], alveolar number, and alveolar surface area (excluding all conducting airways and blood vessels > 10 µm diameter). Mean linear intercept (MLI) was calculated from the H&E stained lung sections by dividing the total length of a line (in micrometers) drawn across the entire field by the total number of alveolar intercepts encountered along the length of the line. Septal count (secondary crests or septae) and septal length were assessed in elastin stained lung sections. The septal length was measured from the base of the primary septum to the tip of the secondary septum and expressed in µm. Lung morphometry assessments were performed by an experienced investigator blinded to the treatment groups by Aperio imaging software (Leica Biosystems, Buffalo Groove, IL).

### KI67 immunohistochemistry

Antigen retrieval was performed on paraffin-embedded sections by heating in citrate buffer (pH-6.0) for 20 min. Slides were washed in PBS and incubated with 2% goat non-immune serum-2% BSA to block nonspecific binding. Lung slides were incubated with the primary antibody for Ki67 (rabbit anti-Ki67, LabVision, Fremont, CA) at 1:250 dilution for 30 min; the secondary antibody and DAB staining kit were used per manufacturer's protocol (Dako Envision-HRP-DAB; Carpinteria, CA). Nonspecific IgG and omission of primary antibody acted as controls for staining specificity. Lung sections were assessed by manual counting of the digitalized slide for Ki67 positive cells at a magnification of 400× (56,000 µm^2^; 20 images per slide; 5 sections/animal; 6 mice/group) by Aperio software. The intensity of brown staining varied from cell to cell. Any degree of brown nuclear staining was identified as a Ki67 positive cell. Cytoplasmic staining was not counted as a Ki67 + cell. Masking was observed to avoid bias in the quantification of Ki67 staining. Staining results were expressed as the number of Ki67 positive cells per HPF.

### Statistical analysis

#### Sample size

The sample size was calculated based on the 'resource equation' method [23] as it was not possible to assess effect size or standard deviation from preliminary data for calculation. We aimed to find any level of differences among the groups. 'E", the degree of freedom of Analysis of Variance (ANOVA) (Total number of animals − Number of groups) was calculated, based on the number of animals (36) and number of groups (6) (36 − 6 = 30) (for gene expression analysis). E of < 10 indicates an increase in the number of animals per group is required, and E > 20 would suggest that adding more animals would not increase the chance of getting a significant result.

All data were expressed as mean ± standard deviation (SD) with *n* representing the number of animals studied (N = 6 in each group). Normal distribution of data was confirmed before analysis by student's t-test and ANOVA. Categorical data were analyzed by the Fisher Exact test. P values were calculated based on students’ test of the replicate 2-∆∆C(t) values for each gene in the control group and the treatment group. ANOVA was performed on 2-∆∆C(t) values to compare differences between the groups. Fisher's post-hoc test analyzed protein expression among the groups.

#### Principal component analysis (PCA)

Principal component analysis, a dimensionality reduction technique, was performed to explain the variance–covariance structure in gene expression data among the groups. We assessed the suitability and the adequacy of the dataset for PCA before analysis. The Kaiser–Meyer–Olkin (KMO) measure of sampling adequacy was 0.73. Bartlett's test of sphericity was statistically significant (p < 0.0005), indicating that the dataset is suitable for data reduction techniques such as PCA or factor analysis. The assumption of normality was met by the rank transformation of data and homogeneity of variance confirmed by the Levene test (P = 0.747). A *p*-value of < 0.05 was considered significant.

## Results

### Cell cycle gene expression at four weeks

Cell cycle gene expression in the 40%O_2_ (Table 1) and 100%O_2_ (Table 2) is compared to the control group (21%O_2_). The fold change in gene expression in the control group is normalized to 1.0, and the fold change in the other two oxygen groups (40%O_2_ and 100%O_2_) is expressed as relative to the control group. Genes are considered overexpressed for a fold change of ≥ 2.0 fold; and under-expressed for fold changes of ≤ 0.5. Of the 84 cell cycle genes analyzed, 13 genes (16%) in the 100%O_2_ group were downregulated (FC ≤ 2; Table 2) compared to 4 genes (5%) in the 40%O_2_ group at four weeks (Table 1) (p < 0.01; Fisher's exact test). Ten genes (12%) in the 100%O_2_ group were overexpressed (FC ≥ 2.0, Table 2) compared to seven (8%) in the 40%O_2_ groups at four weeks (Table 1). Overall, significantly more genes were either over or under-expressed in the 100%O_2_ group than 40%O_2_ group at four weeks (23/84–100%O_2_ *vs.* 11/84–40%O_2_; p < 0.05, Fisher's exact test). *Cdkn1a* (p21), a cyclin-dependent kinase inhibitor, *Ccna2*, *Mki67*, a marker of cell proliferation, and *Ccnb2* were significantly downregulated (p < 0.05) in the 100%O_2_ group at four weeks (Table 2) following short-term perinatal oxygen exposure.

**Table 1:** Cell cycle gene expression by real-time PCR array in lung homogenate of mice at 4- and 12-weeks of age exposed to 30 min of 40%O_2_ within 6 h of birth

Gene symbol	Gene description	Fold change in gene expression
*40%O*_*2*_* Group—4 weeks (Control Group—21%O*_*2*_* 4 weeks)*		
Over**-**expressed genes		
Cdk4	Cyclin-dependent kinase 4	4.26 (0.01–16.5)
Dst	Dystonin	4.34 (0.01–15.4)
Msh2	MutS homolog 2 (*E. coli*)	2.64 (0.01–9.35)
Psmg2	Proteasome assembly chaperone 2	3.28 (0.01–10.3)
Rbl1	Retinoblastoma-like protein 1	2.56 (0.01–7.06)
Shc1	Src homology 2 transforming protein C1	2.21 (0.01–5.09)
Trp63	Transformation related protein p63	2.37 (0.01–5.67)
Under-Expressed Genes		
Ccnb1	Cyclin B1	0.36 (0.01–1.04)
Ccnb2	Cyclin B2	0.50 (0.19–0.82)
Gpr132	G protein-coupled receptor 132	0.15 (0.01–0.76)
Mcm3	Minichromosome maintenance complex-3	0.17 (0.01–0.70)
*40%O*_*2*_* Group—12 weeks (Control Group—21%O*_*2*_* 12 weeks)*		
Over-expressed genes		
Ccnb1	Cyclin B1	5.9 (0.01–10.75)
Trp63	Transformation related protein p63	4.16 (0.01–11.59)
Under-expressed genes		
Abl1	Non-receptor tyrosine protein kinase	0.28 (0.01–0.67)

Genes were considered over-expressed (Fold Change ≥ 2.0) or under-expressed (Fold Change ≤ 0.5) relative to the control group (21%O_2_). The fold change (FC) in gene expression in 21%O_2_ group = 1.0; values expressed as FC with 95% confidence intervals

Housekeeping genes: *Gapdh* (Glyceraldehyde-3-phosphate dehydrogenase), *Hprt* (Hypoxanthine guanine phosphoribosyl transferase) & *Actb* (Actin, beta); N = six mice / group, each time-point

**Table 2:** Cell cycle gene expression by real-time PCR array in lung homogenate of mice at 4- and 12-weeks of age exposed to 30 min of 100%O_2_ within 6 h of birth

Gene symbol	Gene description	Fold change in gene expression
*100%O*_*2*_* Group—4 weeks (Control Group—21%O*_*2*_* 4 weeks)*		
Over-expressed genes		
Cdk4	Cyclin-dependent kinase 4	4.07 (0.01–15.8)
Dst	Dystonin	3.45 (0.001–12.3)
Mre11a	Meiotic recombination 11 homolog A	2.06 (0.001–4.38)
Mdm2	mouse 3T3 cell double minute 2	2.01 (0.54–3.49)
Msh2	MutS homolog 2 (E. coli)	2.91 (0.01–10.32)
Npm2	Nucleoplasmin 2	2.31 (0.02–4.62)
Psmg2	Proteasome assembly chaperone 2	3.28 (0.01–10.3)
Rbl1	Retinoblastoma-like protein 1	2.70 (0.01–7.56)
Shc1	Src homology 2 transforming protein C1	2.52 (0.01–5.85)
Slfn1	Schlafen 1	2.01 (0.51–3.45)
Under-expressed genes		
Ak1	Adenylate kinase 1	0.34 (0.01–0.68)
Abl1	Non-receptor tyrosine protein kinase	0.31 (0.01–0.98)
Ccna2	Cyclin A2	0.35 (0.14–0.57)*
Ccnb1	Cyclin B1	0.08 (0.001–0.31)
Ccnb2	Cyclin B2	0.38 (0.13–0.64)*
Cdkn1a	Cyclin-dependent kinase inhibitor 1A	0.50 (0.31–0.69)*
Gpr132	G protein-coupled receptor 132	0.32 (0.001–1.61)
Mcm3	Minichromosome maintenance complex-3	0.17 (0.001–0.71)
Mki67	Antigen identified by monoclonal antibody Ki-67	0.39 (0.21–0.58)*
Ppm1d	Protein Phosphatase 1D	0.28 (0.001–0.97)
Ran	RAs-related nuclear protein	0.13 (0.01–0.62)
Rbl2	Retinoblastoma-like protein-2	0.22 (0.01–0.81)
Sfn	Stratifin	0.38 (0.001–0.94)
*100%O*_*2*_* Group—12 Weeks (Control Group—21%O*_*2*_* 12 weeks)*		
Over-expressed genes		
Atm	Ataxia Telangiectasia Mutated	2.4 (0.01–7.38)
Ccnb1	Cyclin B1	2.55 (0.001–6.75)
Ccnb2	Cyclin B2	3.38 (0.001–11.3)
Ccnc	Cyclin C	2.28 (0.01–7.15)
Trp63	Transformation related protein p63	2.93 (0.01–8.79)
Under-expressed genes		
Abl1	Non-receptor tyrosine protein kinase	0.33 (0.01–0.88)
Inha	Inhibin alpha	0.52 (0.32–0.73)*
Skp2	S-phase kinase-associated protein 2 (p45)	0.62 (0.46–0.79)*

Genes were considered over-expressed (Fold Change ≥ 2.0) or under-expressed (Fold Change ≤ 0.5) relative to the control group (21%O_2_). The fold change (FC) in gene expression in 21%O_2_ group = 1.0; values expressed as FC with 95% confidence intervals

Housekeeping genes: *Gapdh* (Glyceraldehyde-3-phosphate dehydrogenase), *Hprt* (Hypoxanthine guanine phosphoribosyl transferase) & *Actb* (Actin, beta); N = six mice / group, each time-point

*p < 0.01 *vs* control group

### Cell cycle gene expression at 12 weeks

Gene expression data at 12 weeks are presented in Table 1 (40%O_2_) and Table 2 (100%O_2_). The majority of the cell cycle genes in the 40%O_2_ and 100%O_2_ groups recovered within ± twofold change of the 21%O_2_ groups at 12 weeks. Two cell cycle genes (*Inha* and *Skp2*) were significantly under-expressed in the 100%O_2_ group at 12 weeks (Table 2).

### Principal component analysis of gene expression data

There were no significant differences in cell cycle gene expression (2-∆∆C(t) values) among the three groups at four weeks (p = 0.91) or 12 weeks (p = 0.74) by ANOVA.

### PCA analysis at 4 weeks

**Fig. 1:**
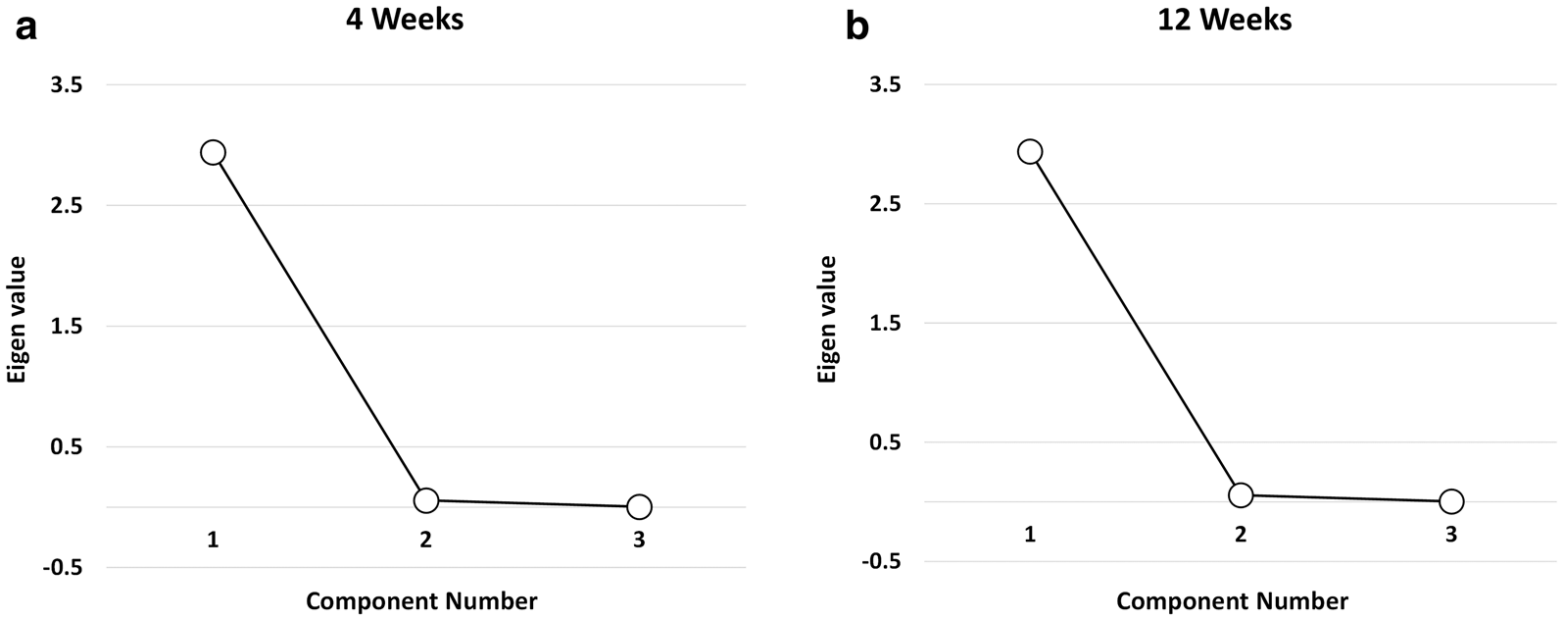
Principal Component Analysis (PCA). The Scree plot demonstrates the Eigenvalues of the principal components in the PCA analysis. The PCA presents one component with an eigenvalue of > 1, which explains 98.03% of the total variation in cell cycle gene expression at 4 weeks (**a**). Similarly, one component with an eigenvalue of > 1 illustrates 97.92% of the total variation in cell cycle gene expression at 12 weeks (**b**)

The proportion of variance accounted for by the PCA was 96.3, 99.1, and 98.7% for 21%O_2_, 40%O_2,_ and 100%O_2_ groups respectively in 4-week-old mice. A scree plot (Fig. 1) displays the eigenvalues on the y-axis and the number of components on the x-axis. PCA revealed one component with an eigenvalue greater than one, explaining 98.03% of the total variance in gene expression (Fig. 1a). The leveling of the slope indicated that including components 2 and 3 would not offer additional benefits, and hence one component was retained for further analysis. The component matrix for the three groups at four weeks is consistent with strong loadings for 21%O_2_ (0.981), 40%O_2_ (0.995), and 100%O_2_ (0.994) (Table 3). The results indicate that the three oxygen groups themselves are highly correlated, suggesting the absence of variance in gene expression among the three oxygen groups.

**Table 3:** Principal component analysis (PCA) correlation matrix

Component 1	
Correlation in gene expression among groups (4-week old mice)	
21%O_2_ Group	0.981
40%O_2_ Group	0.995
100%O_2_ Group	0.994
Correlation in gene expression among groups (12-week old mice)	
21%O_2_ Group	0.981
40%O_2_ Group	0.997
100%O_2_ Group	0.991

Cell cycle gene expression in whole lung homogenate in 4- and 12-week-old mice following short-term (30 min) of perinatal oxygen exposure soon after birth. The principal component analysis revealed one component with Eigenvalue > 1. The PCA component matrix demonstrates a high correlation between each variable in 4- and 12-week-old mice

### PCA analysis at 12 weeks

The proportion of variance accounted for by the PCA was 96.3%, 99.3%, and 98.2% for 21%O_2_, 40%O_2,_ and 100%O_2_ groups respectively in 12-week-old mice. PCA revealed one component with an eigenvalue greater than one, explaining 97.92% of the total variance in gene expression (Fig. 1b). The Steep drop after component one, followed by leveling off the slope (component 2 and 3), indicate that we retain component one for matrix analysis. The component matrix for the three groups is consistent with strong loadings for 21%O_2_ (0.981), 40%O_2_ (0.997), and 100%O_2_ (0.991) in 12-week old mice (Table 3). The results indicate that the three oxygen groups are highly correlated, suggesting an absence of variance in gene expression among the three oxygen groups.

### Cell cycle protein analysis

Cdkn1a (p21) and Trp6 protein were not significantly different among the groups at four weeks and 12 weeks (Table 4). Lung elastin was decreased considerably in the 100%O_2_ group compared to the 21%O_2_ group at four weeks (Table 4, p < 0.05; ANOVA). However, elastin levels in the lung were similar in the three oxygen groups at 12 weeks. Ki67 protein was significantly higher in the 40%O_2_ group than 21%O_2_ and the 100%O_2_ group at four weeks (Table 4, p < 0.05 *vs.* 21%O_2_ & 100%O_2_, ANOVA). However, Ki67 levels were not different among the groups at 12 weeks. Similarly, Cyclin B1 was significantly higher in the 40%O_2_ group than 21%O_2_ at four weeks (Table 4, p < 0.05 *vs.* 21%O_2,_ ANOVA). However, cyclin B1 in the lung was not different among the groups at 12 weeks.

**Table 4:** Protein expression of selected cell cycle proteins and elastin in lung homogenates measured by enzyme immunoassay (EIA) in mice at 4 and 12 weeks

Protein	4 Weeks			12 Weeks		
	21% O_2_	40% O_2_	100% O_2_	21% O_2_	40% O_2_	100% O_2_
CCNB1	27.32 ± 3.54	34.60 ± 6.29*	29.08 ± 3.26	28.29 ± 3.54	21.59 ± 4.73	25.04 ± 4.65
MKI67	17.05 ± 2.98	25.16 ± 1.4*^†^	19.24 ± 2.38	26.63 ± 5.67	22.35 ± 4.15	25.51 ± 7.39
CDKN1A (p21)	12.60 ± 0.31	12.58 ± 0.28	12.25 ± 0.28	14.33 ± 3.68	12.89 ± 0.30	12.71 ± 0.08
Trp63	2.46 ± 2.71	3.55 ± 2.51	0.66 ± 0.15	1.67 ± 0.13	1.48 ± 1.01	1.37 ± 0.66
ELN	1.14 ± 0.37	0.96 ± 0.22	0.48 ± 0.24*	0.23 ± 0.20	0.57 ± 0.40	0.62 ± 0.42

The mice were exposed to 30 min of oxygen exposure (21%O_2_, 40%O_2,_ or 100%O_2_) within six hours after birth

Values were expressed as mean ± SD (N = 6 in each group, each time point)

*CCNB1* cyclin B1, *Ki67* marker of proliferation Ki-67, *CDKN1A (p21)* cyclin-dependent kinase inhibitor 1A, *Trp63* tumor protein 63, *ELN* elastin, Cell cycle proteins and elastin expressed as pg/µg lung protein

*p < 0.05 *vs.* 21%O_2_ group

^†^p < 0.05 *vs.* 100% O_2_ group (Fisher's Post-Hoc test, ANOVA)

### Ki67 immunostaining for cell proliferation

**Fig. 2:**
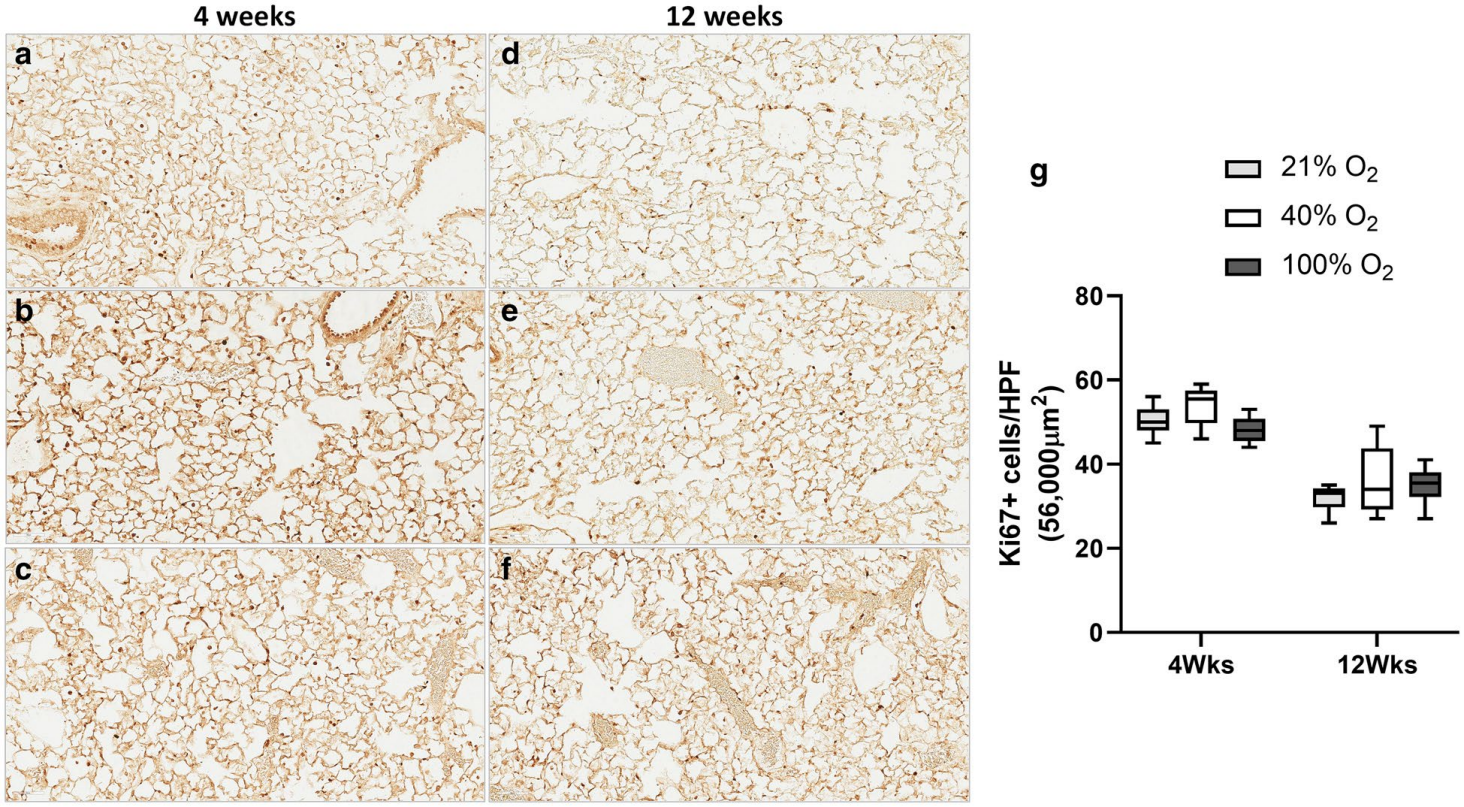
Ki67 Immunostaining. Staining for Ki67, a marker of cellular proliferation was performed on lung sections at four weeks (**a** 21%O_2_, **b** 40%O_2_ & **c** 100%O_2_) and 12 weeks mice (**d** 21%O_2_, **e** 40%O_2_ & **f** 100%O_2_) following neonatal oxygen exposure (Scale bar: 60 µm). Ki67 positive cells for nuclear staining was calculated at 400 × resolution (56,000 µm^2^ area) in all the groups (**g** five sections/mice; 6 mice/group; 21%O_2_—grey bars; 40%O_2_—white bars; 100%O_2_—black bars). The number of Ki67 positive cells demonstrated a significant correlation over time but not with the oxygen groups (p < 0.001, Two-way ANOVA). On multiple comparisons, there was no significant difference among the oxygen groups

Nuclear staining for Ki67 was assessed by immunostaining at four weeks and 12 weeks of age (Fig. 2). Nuclear staining was quantified by counting Ki67 positive cells in the high power field. There was a significant interaction of the number of Ki67 positive cells over time (p < 0.001; Two-way ANOVA) but not for the oxygen groups. On multiple comparisons, there was no difference in the number of Ki67 positive cells among the three oxygen groups, suggesting that cell proliferation is not significantly different among the groups in mice at four weeks and 12 weeks (Fig. 2g).

### Lung morphometry

**Fig. 3:**
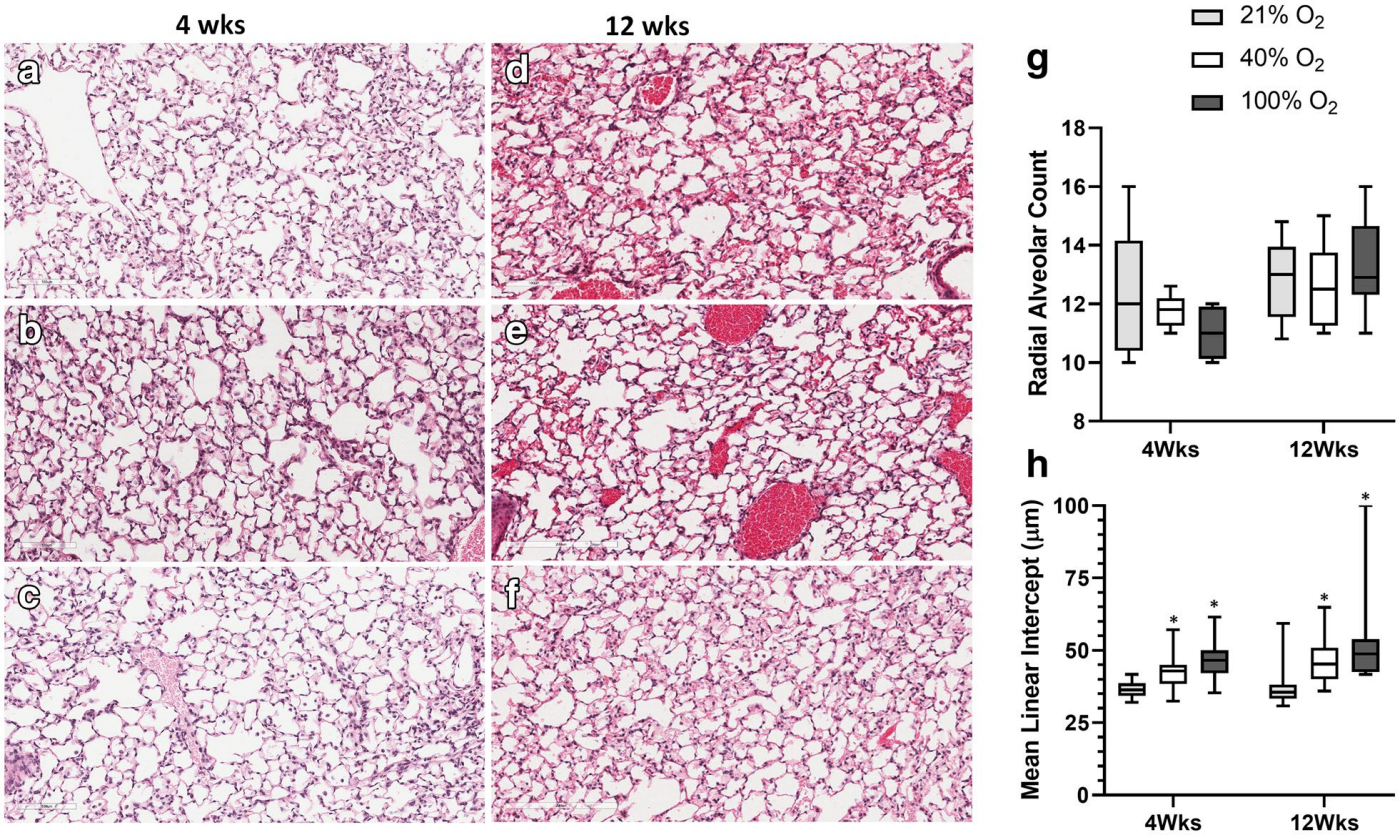
Lung histopathology. Lung sections were assessed by H&E staining in all the groups at four weeks (**a** 21%O_2_, **b** 40%O_2_ & **c** 100%O_2_) and 12 weeks of age (**d** 21%O_2_, **e** 40%O_2_ & **f** 100%O_2_) following neonatal oxygen exposure. Lungs did not demonstrate gross evidence of alveolar simplification (Scale bar: 100 µm). Radial alveolar count (RAC) (**g**) and mean linear intercept (MLI) (**h**) was estimated in all the groups (21%O_2_—grey bars; 40%O_2_—white bars; 100%O_2_—black bars; 400 × resolution). RAC was not significantly different in the groups at four weeks and 12 weeks in mice (**g**). However, MLI demonstrated a significant interaction concerning oxygen groups (p < 0.001; Two-way ANOVA). On multiple comparisons, exposure to 100%O_2_ soon after birth resulted in a significant increase in MLI at 4 and 12 weeks of age in mice (*p < 0.001 *vs.* 21%O_2_ & 40%O_2_ groups, Two-way ANOVA). (n = 6/group, each time-point)

**Fig. 4:**
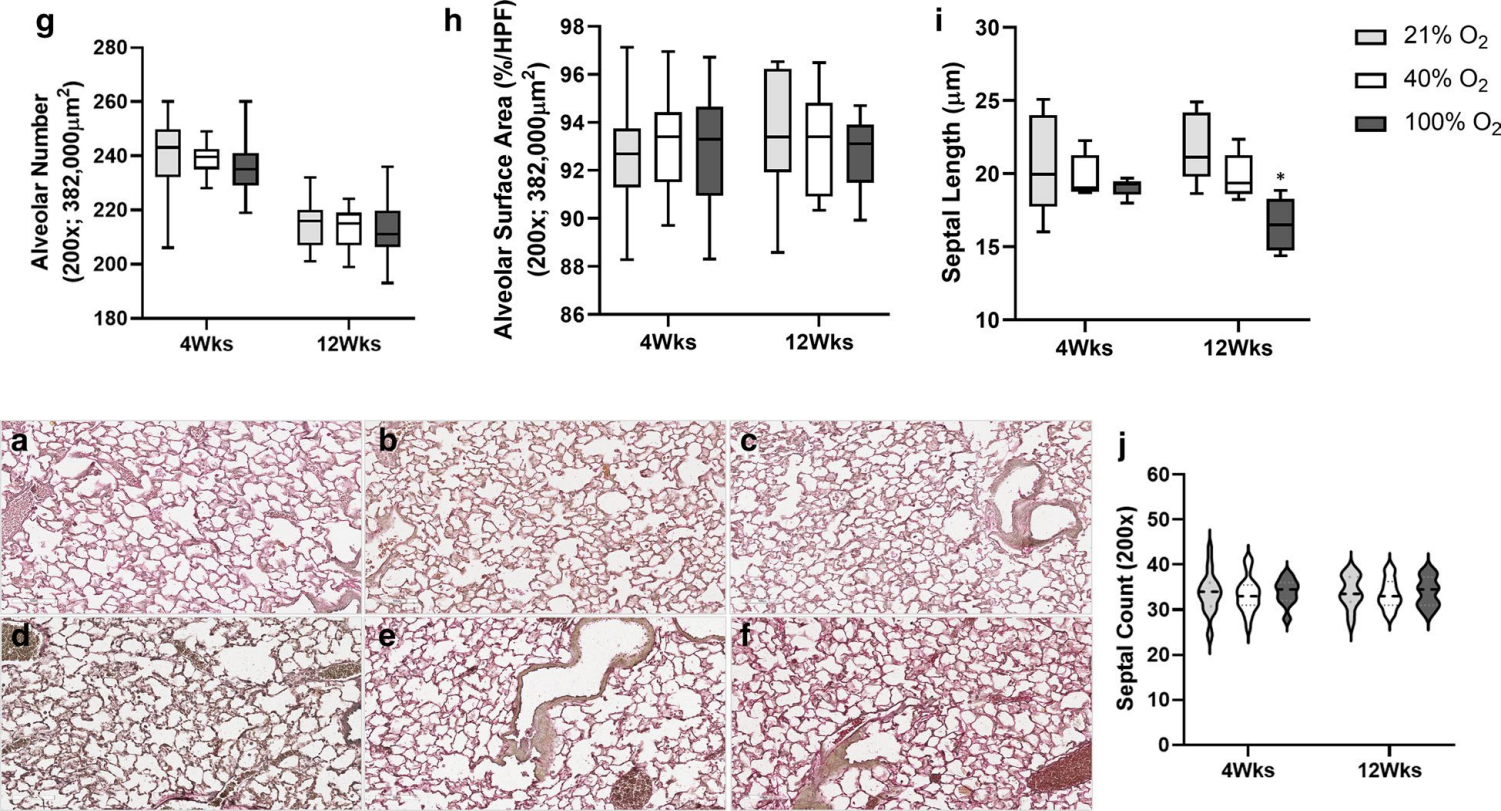
Elastin Staining & Lung Morphology. Lungs were assessed by elastin staining in all groups at four weeks (**a** 21%O_2_, **b** 40%O_2_ & **c** 100%O_2_) and 12 weeks (**d** 21%O_2_, **e** 40%O_2_ & **f** 100%O_2_) of age following neonatal oxygen exposure (Scale bar: 100 µm). Alveolar number (**g**), alveolar surface area per high power field (HPF) (850 × 450 µm; 382,000 µm^2^) (**h**), septal length (**i**), and septal count (**j**) were assessed at 200 × resolution. There was no difference in alveolar number, alveolar surface area, and septal count among the three oxygen groups at both time points. Septal length demonstrated a significant interaction among the three oxygen groups over time (p < 0.01, Two-way ANOVA). On multiple comparisons, exposure to 100%O_2_ soon after birth resulted in a significant reduction in the length of the secondary septae at 12 weeks of age in adult mice (*p < 0.01 *vs.* 21%O_2_ & 40%O_2_ groups, Two-way ANOVA). (n = 6/group, each time-point)

Histopathology, as assessed by H&E staining of lung sections, demonstrated no gross evidence of alveolar simplification among the groups at four weeks (Fig. 3a–c) and 12 weeks of age (Fig. 3d–f). The radial alveolar count was not significantly different among the groups at four weeks and 12 weeks in mice (Fig. 3g). However, MLI demonstrated a significant interaction concerning oxygen groups (p < 0.001; Two-way ANOVA). On multiple comparisons, exposure to 100%O_2_ soon after birth resulted in a significant increase in MLI at 4 and 12 weeks of age in adult mice (*p < 0.001 *vs.* 21%O_2_ & 40%O_2_ groups, Two-way ANOVA), suggesting subtle changes of impaired alveolarization following 30 min of oxygen exposure in the perinatal period. Morphometry of the lung was further detailed by assessing the alveolar number, alveolar surface area, septal count, and septal length on elastin stained sections of the three oxygen groups at four weeks (Fig. 4a–c) and 12 weeks (Fig. 4d–f). There was no difference in alveolar number (Fig. 4g), alveolar surface area (Fig. 4h), and septal count (Fig. 4j) among the three oxygen groups at both time points. However, septal length demonstrated a significant interaction among the three oxygen groups over time (p < 0.01, Two-way ANOVA). On multiple comparisons, exposure to 100%O_2_ soon after birth resulted in a significant reduction in length of the secondary septae at 12 weeks of age in adult mice (*p < 0.01 *vs.* 21%O_2_ & 40%O_2_ groups, Two-way ANOVA).

## Discussion

Exposure to a high concentration of oxygen in the neonatal period impairs lung growth and is a major contributing factor in the development of BPD. Oxygen concentrations that can be safely administered at resuscitation and subsequently in the postnatal management of premature infants at risk for BPD and ROP are uncertain at best. Studies on the effects of oxygen on gene expression are particularly relevant in newborns. Changes in gene expression at critical times of development can have long-lasting consequences with changes in lung structure and function [24]. Neonatal hyperoxia markedly inhibits lung epithelial cell proliferation [25] during the period of maximum alveolarization of the lung [26], implying alterations in gene expression, particularly relating to the regulation of cell cycle [27]. We studied the long-term effects of exposure to clinically relevant oxygen concentrations, including 100%O_2_ after birth on cell cycle gene expression and lung structure in adult mice.

Exposure to oxygen for 30 min in the perinatal period resulted in the downregulation of several cell cycle genes in 4-week-old mice, particularly in the 100%O_2_ group. An increased number of cell cycle genes were downregulated in the 100%O_2_ group compared to the 40%O_2_ group at four weeks, perhaps suggesting that oxygen effects on gene expression may be dose-dependent. However, gene expression among the groups was not different at 12 weeks of age. Among the 84 genes studied by array analysis, none of the genes in the 40%O_2_ group was statistically significant from room air controls either at four weeks or at 12 weeks. Microarray experiments allow simultaneous rapid assessment of hundreds of genes; however, several factors affect their reliability [28], requiring confirmation by RT-PCR or protein analysis. There were no significant differences noted in gene expression either by ANOVA or by PCA analysis. The scree plot retained only one principal component, and the component matrix demonstrated a high correlation of greater than 98% among the groups at both four weeks and 12 weeks. The high correlation among the groups suggests that the concentration of oxygen administered in the perinatal period had minimal effect on gene expression at four weeks and 12 weeks of age in adult mice. Species differences, biological variability, and array procedure may have affected the significant differences noted in the expression of several genes in the 100%O_2_ groups.

Ki67, a marker gene for cell proliferation, was significantly downregulated in the 100%O_2_ group; however, the Ki67 protein expression was not different from 21%O_2_ at 4 and 12 weeks. A significantly higher Ki67 protein in the lung was noted in the 40%O_2_ group despite the lack of differences in gene expression at four weeks. Ki67 expression in tissues is highly heterogeneous and is influenced by the stage of the cell cycle and the time spent in G0 [29]. Higher levels of Cyclin B1, a regulator of the cell cycle, suggest that elevation of Ki67 in lung tissue may be related to cell cycle changes [30]; however, the functional significance of Ki67 protein expression is often unclear [31]. Additionally, Ki67 immunostaining did not demonstrate differences in Ki67 + cells among the groups at both four weeks and 12 weeks. However, a significant difference in Ki67 staining over time may reflect active cell proliferation from lung development at four weeks. During the active stage of lung development in the postnatal lung, downregulation of p21 is essential for effective DNA synthesis, as cells expressing low levels of p21 progress through cell cycle upon release from S-phase arrest [32], facilitating lung recovery in developing mice. Higher expression of p21 was noted following exposure to 95%O_2_ for 24 or 48 h, suggesting overall inhibition of cell cycle progression [33]. The absence of any differences in p21 expression may indicate no significant differences in lung development. However, the study is limited by the lack of p21 measurement closer to hyperoxia exposure after birth.

Mice lung is composed of large sacculi by postnatal day 4 (P4), formed by branching morphogenesis; and alveolarization occurs from P4 to P21 with new septa formation from immature pre-existing septa [34, 35]. Lifting-up of new septae (also called 'secondary septae' or 'secondary crests') from pre-existing mature septa occurs from P14 into early adulthood and is virtually complete by P36 [34, 35]. Alveoli formation occurs at a faster pace from P4 to P14 and at a slower rate after that. The development of a single layer capillary network in the septa coincides with not only the completion of alveolarization but also microvascular maturation [34]. In our study, 4-week old mice are in the final phase of alveolarization corresponding to young adulthood, with alveolarization virtually complete in adult mice at 12 weeks.

A significant reduction in lung elastin in the 100%O_2_ group at four weeks indicates the enduring effects of even brief exposure to high oxygen concentration. Even though elastin measurements in lung homogenates were not different among the groups at 12 weeks, MLI was significantly higher in the 100%O_2_ groups at both 4 and 12 weeks of age. Septal length, a measure of secondary crest formation, was significantly different among the three oxygen groups, with a significant decrease in septal length in the 100%O_2_ group compared to 21%O_2_ and 40%O_2_ group adult mice. Not all measurements of lung morphometry demonstrate lung impairment, as differences in lung development may be challenging to determine from single measurements; however, septal thickness and MLI are sensitive objective tools of lung morphometry. Taken together, exposure to 30 min of 100%O_2_ administration may modify secondary crest maturation, alter lung elastin, and remodel lung development.

The postnatal alveolar formation is the most important and the least understood phase of lung development. Tightly regulated differentiation of alveolar fibroblasts towards a myogenic or a lipogenic phenotype is relevant to the septal formation [36]. This is driven to some extent by Platelet-Derived Growth Factor Receptor (PDGFRα). Postnatal PDGFRα is crucial in regulating elastogenesis [37]. The stability of the extracellular matrix involving collage and elastin is perturbed during the arrested alveolarization of the developing mouse lung exposed to hyperoxia [38]. We have shown that prolonged hyperoxia in the postnatal period results in permanent alveolar simplification in mice [39, 40]. Hyperoxia exposed cells undergo both apoptotic and nonapoptotic cell death. An increase in apoptosis from hyperoxia during a critical period of lung development may be an essential factor in impaired lung growth and remodeling [41]. Clinical studies have shown that resuscitation with high oxygen concentration (90%O_2_) results in higher oxidative stress, inflammation, and bronchopulmonary dysplasia than resuscitation with 30%O_2_ in premature infants [21].

Newborn mice at birth are equivalent to 26-week gestation human infant and 30 min of oxygen, in mice is similar to oxygen exposures premature infants experience at birth. As randomization was not based on littermates from different litters, bias might have been introduced due to possible litter effects. If 30 min of exposure would not affect gene expression, then probably anything less is less likely to affect newborn mice. In humans, the duration of pure O_2_ breathing needed to induce oxygen toxicity is not known. However, supraphysiologic levels of O_2_ prolong cellular dysfunction with increasing morbidity and mortality [42, 43]. Hyperoxic reoxygenation affects multiple signaling pathways in the lung that regulate cell growth, DNA repair, and survival [44]. An exposure period of 6–24 h is associated with clinical and histologic signs of lung injury in humans [45, 46]. The effects of prolonged hyperoxia on gene expression is well studied [47]; however, the effects of shorter duration (30 min to 6 h) needs further exploration. We analyzed gene expression in the whole lung instead of specific lung cells such as alveolar epithelial or airway epithelial cells, which is a limitation. The lack of gene expression, protein analysis, and oxidant injury markers closer to hyperoxia exposure could have been relevant to the study. The study is limited by the lack of exposure to oxygen at birth that usually occurs in infants requiring resuscitation. Early hyperoxia may induce changes in histone signatures in gene expression, altering vascular patterns in infants with BPD [48]. The timing, duration, and severity of hyperoxia relative to resuscitation need critical exploration. In a National Collaborative Perinatal Project, a slightly higher risk of cancer was noted in children exposed to > 3 min of oxygen at birth [49]. Even though 30 min of 100%O_2_ by itself does not produce changes in cell cycle gene expression, factors such as intrauterine or postnatal infection or respiratory depression at birth that may affect resuscitation and oxygen administration needs to be studied further. The development of lung injury during hyperoxia exposure is a complex process with the expression of several genes essential in the adaptive response to hyperoxia, including apoptosis, cytokine production, and extracellular matrix repair [33]. We focused on cell cycle gene expression and its impact on cell proliferation and lung development, one of the critical components of responses to hyperoxia.

## Conclusions

The study demonstrates that short-term exposure to high oxygen concentrations leads to subtle changes in lung development that may affect alveolarization. These changes are related explicitly to secondary crest formation that may result in alteration in lung elastin. The effects of resuscitation with high oxygen concentrations may have a significant impact on lung development and long-term outcomes such as BPD in premature infants. Alveolar development resulting from the formation of the secondary crest and microvasculature maturation is being explored. The role of oxygen in alveolar development and its relationship to crest formation and lung elastin is not clear. The data generated from the study is not only a step in understanding lung development in the context of oxygen resuscitation but also its long-term impact, specifically in the lung. The information may be useful in term and premature newborns at resuscitation and beyond, wherein oxygen is routinely used in managing these infants.

## Data Availability

The datasets used and analyzed during the current study are available from the corresponding author on reasonable request.

## Abbreviations

PCA: Principal Component Analysis
BPD: Bronchopulmonary Dysplasia
ROP: Retinopathy of Prematurity
